# Study on supplemental test to improve the detection of bovine tuberculosis in individual animals and herds

**DOI:** 10.1186/s12917-021-02839-4

**Published:** 2021-03-31

**Authors:** Paulo Alex Machado Carneiro, Eliomar de Moura Sousa, Rinaldo Batista Viana, Bruno Moura Monteiro, Aline do Socorro Lima Kzam, Damazio Campos de Souza, Anderson Silva Coelho, José Dantas Ribeiro Filho, Ricardo Spacagna Jordao, Maria Regina Madruga Tavares, John B. Kaneene

**Affiliations:** 1 Amazonas State Federal Institute of Science and Technology, Manaus East Zone Campus (IFAM/ CMZL), Manaus, Brazil; 2 Center for Comparative Epidemiology, College of Veterinary Medicine, Michigan State University, East Lansing, USA; 3 Independent researcher, Belém, Brazil; 4 Graduate Program in Animal Reproduction, Institute of Health and Animal Production, Federal Rural University of the Amazon, Belem, Brazil; 5 Research Group on Andrology, Artificial Insemination, Health and Genetic Improvement of Cattle and Buffaloes, Amazon, Brazil; 6 Federal University of Vicosa, Vicosa, Brazil; 7 Instituto Biologico, São Paulo, Brazil; 8 Institute of Mathematics and Natural Sciences, Federal University of Pará, Belem, Brazil

**Keywords:** Bovine tuberculosis, *Mycobacterium bovis*, Tuberculin test, ELISA, Sensitivity, Specificity

## Abstract

**Background:**

Bovine tuberculosis (bTB), is a worldwide disease caused by *Mycobacterium bovis* (*M. bovis*). The success of bTB eradication and control programs is based on early detection and the removal of reactors from a herd thus routine testing and cull strategy have been applied globally. Since the late nineteenth century, the Tuberculin Skin Test (TST) has been the primary antemortem test available to support bTB eradication campaigns. Due to the TST limitations in terms of Se and Sp, the credibility of the diagnosis is frequently questioned given the occurrence of false-positive and false-negative reactions, therefore, it is necessary to confirm reactive animals using other methods, ensuring the reliability of the diagnosis. The aim of this study was to evaluate the sensitivity and specificity of a multiplex enzyme-linked immunosorbent assay (ELISA) relative to the tuberculin test used for the diagnosis of tuberculosis in cattle in Brazil.

**Results:**

Lack of agreement between comparative cervical tuberculin test and ELISA IDEXX TM was observed. The 2 animals positive on the comparative cervical tuberculin test did not react at the ELISA IDEXX TM and 22 negative reactors by comparative cervical tuberculin test were positive by the ELISA IDEXX TM. The ELISA IDEXX TM showed sensitivity that is significantly lower than the official screening test the single cervical tuberculin. ELISA IDEXX TM also detected infected animals and herds undetected by the comparative cervical tuberculin test. The parallel use of comparative cervical tuberculin test and ELISA IDEXX TM increased sensitivity and the feasibility bTB screening.

**Conclusions:**

The results obtained here suggest that the ELISA IDEXX TM may be a supplemental test for the detection of *Mycobacterium bovis* infection in regions without routine testing and slaughter, where the disease generally progresses to more advanced stages and antibody responses are likely to be more prevalent. Evidence to support the validation of the ELISA IDEXX™ as a supplemental test for bTB eradication programs was provided.

**Supplementary Information:**

The online version contains supplementary material available at 10.1186/s12917-021-02839-4.

## Background

Bovine tuberculosis (bTB), is a worldwide disease caused by *Mycobacterium bovis* (*M. bovis*) and affects mainly cattle but also several other species; including humans causing the zoonotic tuberculosis (TB) [1–4]. The disease is difficult to control due to the lack of an effective vaccine, the presence of wildlife reservoirs, and the absence of a diagnostic assay with sufficient sensitivity (Se) and specificity (Sp) to detect sick animals at all stages of infection [4, 5]. The success of bTB eradication and control programs is based on early detection and the removal of reactors from a herd [6] thus routine testing and cull strategy have been applied globally [3]. Therefore, screening-test accuracy is critical to eradication programs. The single intradermal cervical tuberculin (SCT) test; the single intra-dermal comparative cervical tuberculin (CCT) test in Europe; the caudal fold tuberculin (CFT) test in North America, Australia and New Zealand [7]; and the SCT and CFT tests in Brazil [6], are the prescribed tests for international trade [8]. From a practical point of view, the diagnostic performance, the feasibility of execution and practicality of each test, as well as the costs and associated biological risks, should be considered for better strategic use [7].

Since the late nineteenth century, the Tuberculin Skin Test (TST) has been the primary antemortem test available to support bTB eradication campaigns [9, 10]. Advantages of the TST and reasons for its wide use are low costs, high availability, long history of use and, for a long time, the lack of alternative methods to detect bTB [9–12]. On the other hand, this test has many known limitations, including difficulties in performance and result interpretation, need for a second step visit, low degree of standardization, and reduced test accuracy [13].

Due to the TST limitations in terms of Se and Sp, the credibility of the diagnosis is frequently questioned given the occurrence of false-positive and false-negativereactions, therefore, it is necessary to confirm reactive animals using other methods, ensuring the reliability of the diagnosis [5–7, 10]. Research continues into the development of new, more accurate, more sensitive tests, less subject to the individual operative performance and subjective interpretation [14–19]. As the TST are limited to cell-mediated immune responses (CMI) in the early stages of the infection, a serological test with good Se and Sp aiming at the detection of antibodies to *M. bovis* in animals in late stages of the disease, would be a viable complementary technique to improve the detection of bTB [9].

Over the last few years, the potential for use of an antibody assays to detect *M. bovis* infection in cattle is being consolidated [16–25]. The enzyme-linked immunosorbent assay (ELISA) has proven to be useful as ancillary serial (to enhance Sp) and parallel (to enhance Se) tests in several species [26–30]. Moreover, a booster effect on the antibody response caused after injection of tuberculin has been reported and recommended as a strategic option to increase the Se of serological assays [26–28]. The ELISA using MPB83 and MPB70 antigens (IDEXX *M. bovis* Ab Test, IDEXX Laboratories, Westbrook, Maine, United States (US)) is an immune enzymatic assay promising Se and Sp superior to most tuberculosis diagnoses for both primary and supplementary diagnosis in cases of inconclusive results of the diagnosis by simple or comparative cervical tests [12, 16, 31].

The Brazilian guidelines for control and eradication of animal tuberculosis determines intradermal tuberculin testing as the standard method of diagnosing bTB [32]. The primary screening is performed using the CFT test (beef only), and the SCT or the CCT test (dairy and beef). The CCT is also adopted as a confirmatory test. In recent years, epidemiological studies adopting TST were made and were aimed at determining the bTB status in several Brazilian states. The herd prevalence ranged from 0.3 to 7.5% and the animal prevalence ranged from 0.03 to 1.3% [32]. Available data from 2017 regarding cattle organs and/or carcass condemnation in Para state demonstrated that 12,183 carcasses and 837,744 cattle organs were condemned in that year. Lungs (46.83%), kidneys (17.06%), heads (10.11%), livers (9.74%), and intestines (5.59%) were the major condemned organs. TB was the cause of 4.88% of the condemnations [33].

Until 2019, no other ancillary indirect methods for bTB diagnoses were approved by the Brazilian Ministry of Agriculture, Livestock and Food Supply (MAPA). Adoption of complementary field bTB diagnostic tests can improve the detection of infected animals and herds. Due to absence of mandatory test and elimination police the reference standard diagnosis test (culture) is not available, to overcome this obstacle a Bayesian latent class analysis is recommended [31].

To the best of our knowledge, there is no in vivo study comparing the CCT test with ELISA IDEXX™ to evaluate it as a supplemental test for bTB in beef cattle in Brazil. The objective of this study was to determine the Se and Sp of the CCT test and a commercial ELISA multiple test (ELISA IDEXX™), under field conditions using a Bayesian approach in order to provide evidence-based data to support adoption of ancillary tests in beef cattle aiming at the improvement of national bTB eradication programs.

## Results

### Prevalence of tuberculin skin test x ELISA for the diagnosis of bovine tuberculosis in beef cattle

The SCT test (considering only the PPDb values), revealed positive animals in all the studied farms, with higher values on farm 4 followed by farms 3, then 5 and then 2, and with lower values on farm 1 (Table 1).

**Table 1:** SCT (PPDb), CCT and ELISA bTB results in 400 Nelore cattle by farm

Farm	Exam % (n/n)					
	SCT (PPDb)*		CCT**		ELISA***	
	–	+	–	+	–	+
1	96.25 (77/80)	3.75^c^ (3/80)	100 (80/80)	0.0^c^ (0/80)	98.75 (79/80)	1.25^c^ (1/80)
2	92.50 (74/80)	7.50^bc^ (6/80)	100 (80/80)	0.0^bc^ (0/80)	93.75 (75/80)	6.25^bc^ (5/80)
3	86.25 (69/80)	13.75^b^ (11/80)	98.75 (79/80)	1.25^b^ (1/80)	90.0 (72/80)	10.0^b^ (8/80)
4	62.50 (50/80)	37.50^a^ (30/80)	98.75 (79/80)	1.25^a^ (1/80)	91.25 (73/80)	8.75^a^ (7/80)
5	88.75 (71/80)	11.25^bc^ (9/80)	100 (80/80)	0.0^bc^ (0/80)	98.75 (79/80)	1.25^bc^ (1/80)
**TOTAL**	**85.25 (341/400)**	**14.75**^**A**^ **(59/400)**	**99.50 (398/400)**	**0.50**^**C**^ **(2/400)**	**94.50 (373/400)**	**5.50**^**B**^ **(22/400)**

Different uppercase letters between lines and different lowercase lines between columns differed between them (P < 0,0001). *SCT (PPDb) – Simple Cervical Test, **CCT – Comparative Cervical Test, *** ELISA IDEXX *M. bovis*

The CCT test results showed that 2/400 animals were classified as positive (reactors). In the experiment carried out on farms 3 and 4, the CCT test results were similar. Out of 80 tested animals per farm, 1 animal in each of the herds had a positive result, however, the CCT reactive tested animals were not reactive on the ELISA test. On farms 1, 2 and 5, all animals were non-reactive on the CCT test, therefore, only 2/5 herds were considered positive for bTB, according to the CCT testing on this study. However, the results obtained by ELISA IDEXX™ were different from those indicated by the CCT test. A total of 22 out of the 400 animals (5.5%) were considered positive. At the herd-level, all the 5 herds (100%) had at least one positive animal (Table 1).

It was possible to observe that the SCT test had an occurrence of 14.75% (59/400) of positive animals, while the CCT test was 0.50% (2/400) and the ELISA detected 5.50% (22/400) (*P* < 0.0001). Note that the number of animals reacting to the SCT test was greater than the ELISA and CCT tests. Similarly, there was a higher frequency of animals positive in the ELISA compared to the CCT test (Table 1).

When comparing the CCT and SCT tests, it was observed that of the 59 positive tests for SCT, only two were equally positive for CCT (Table 2). In the comparison of the CCT test with the ELISA, the two animals reacting to the CCT were negative to the ELISA, while 22 animals were positive to the ELISA but negative to the CCT (Table 3). Regarding the prevalence of SCT x ELISA, it was possible to indicate that of the 59 animals positive for SCT, only six of them were equally reactive to ELISA. Another 16 animals were positive for ELISA but not reactive to SCT (Table 4).

**Table 2:** Comparison of the results of the CCT x SCT (PPDb) in 400 Nelore cattle

	SCT (PPDb)		TOTAL
	Negative	Positive	
**CCT**			
** Negative**	341 (85.25%)	57 (14.25%)	398 (99.50%)
**Positive**	0 (0.0%)	2 (0.50%)	2 (0.50%)
**TOTAL**	341 (85.25%)	59 (14.75%)	400 (100.0%)

*P* < .0001; Kappa = 0.0564

**Table 3:** Comparison of the results CCT x ELISA in 400 Nelore cattle

	ELISA		TOTAL
	Negative	Positive	
**CCT**			
**Negative**	376 (94.0%)	22 (5.50%)	398 (99.50%)
**Positive**	2 (0.50%)	0 (0.0%)	2 (0.50%)
**TOTAL**	378 (94.50%)	22 (5.50%)	400 (100.0%)

P < .0001; Kappa = − 0.0093

**Table 4:** Comparison of the results SCT x ELISA in 400 Nelore cattle

	ELISA		TOTAL
	Negative	Positive	
**SCT (PPDb)**			
**Negative**	325 (81.25%)	16 (4.0%)	341 (85.25%)
**Positive**	53 (13.25%)	6 (1.50%)	59 (14.75%)
**TOTAL**	378 (94.50%)	22 (5.50%)	400 (100.0%)

P < .0001; Kappa = 0.0739

### Sensitivity and specificity of bTB tests (CCT x ELISA)

In Brazil, the CCT test is considered a confirmatory test, while the SCT test is a screening test for beef cattle. Due to logistical conditions, it was decided to establish a comparative analysis of the Se and Sp of the ELISA against the CCT.

The results of the Bayesian latent class analysis indicated a bTB apparent prevalence of 0.33%. No significant differences were found between the CCT and ELISA tests. The CCT presented lower Se than ELISA but a slightly higher Sp (Table 5).

**Table 5:** Bayesian estimates of Prevalence, Sensitivity, and Specificity of bTB tests with a 95% Confidence Interval

Parameter	Estimative	CI
AP	0.0033	(0.0005; 0.0087)
Se_CCT_	0.7329	(0.5986; 0.8497)
Se_ELISA_	0.8882	(0.8063; 0.9513)
Se_CCT_ – Se_ELISA_	−0.1553	(−0.3049; − 0.0158)
Sp_CCT_	0.9557	(0.9372; 0.9718)
Sp_ELISA_	0.9479	(0.9256; 0.9665)
Sp_CCT_ – SP_ELISA_	0.0078	(−0.0186; 0.0357)

*AP* Apparent Prevalence; *Se*_*CCT*_ Comparative Cervical Test Sensitivity; *Se*_*ELISA*_ ELISA IDEXX™ Sensitivity; *Sp*_*CCT*_ Comparative Cervical Test Specificity; *Sp*_*ELISA*_ ELISA IDEXX™ Specificity

The parallel interpretation of results of the two tests show an increase of Se at herd-level on the official CCT test from 40 to 100% and at animal-level from 0.5 to 6.00% (Tables 6 and 7).

**Table 6:** Single and parallel interpretation of CCT + ELISA IDEXX™ at Herd-level in 400 Nelore cattle

Results	Tests		
	CCT^a^	ELISA^b^	CCT+ ELISA
**Positive**	**40%** (2/5)	**100%** (5/5)	**100% (5/5)**
**Negative**	**60%** (3/5)	**0%** (0/5)	**0% (0/5)**

^*a*^*CCT* Comparative Cervical Test, ^b^ ELISA IDEXX™ *M. bovis*

**Table 7:** Single and parallel interpretation of CCT + ELISA IDEXX™ at Animal-level in 400 Nelore cattle

Results	Tests		
	CCT^a^	ELISA^b^	CCT+ ELISA
**Positive**	**0,50%** (2/400)	**5,50%** (22/400)	**6%** (24/400)
**Negative**	**99,50%** (398/400)	**94,50%** (378/400)	**94%** (376/400)

^a^*CCT* Comparative Cervical Test, ^b^ ELISA IDEXX™ *M. bovis*

## Discussion

This study assessed the performance of the bTB tests routinely used in eradication programs (SCT and CCT tests) and a potential supplemental test (ELISA IDEXX™) under field conditions in Brazil using a Bayesian approach and parallel interpretation of the tests.

ELISA IDEXX™ presented higher Se than CCT testing, even in the absence of the booster effect. The evaluation of experimental diagnostic techniques for the detection of antibodies against *M. bovis* has demonstrated that depending on the epidemiological situation the Se of the antibody detection tests in the absence of the booster effect could be even bigger than the one obtained using official techniques that detect the cell-mediated immune (CMI) response [27, 28]. Recently, under a high bTB prevalence situation, serological tests presented higher Se than official techniques in the absence of a booster effect [17, 29]. In our study, adopting a parallel interpretation the prevalence ranged from 1.25–11.25% within the herds and may explain the higher Se presented by ELISA IDEXX™.

No overlap was found between the 22 animals with a positive ELISA IDEXX™ and the two animals with positive CCT test results, which compared to similar findings of other studies [33–35]. However, in this study six animals (1.50%) reactors on the SCT test were positive on ELISA IDEXX™, which differ from a previous study where no agreement was found between SCT testing and ELISA IDEXX™ [34]. The lack of, or low agreement between, the positive results of the two tests may reflect different elements of the immune response (humoral and cell-mediated immunity) [34, 35]. As TST’s are efficient to detect CMI responses in the early stages of the infection with *M. bovis*, it usually fails to detect chronic stages of the infection [26, 29, 36].

In Brazil, due to the absence of the test and slaughter policy, ranchers usually test their herds to detect bTB only when required by law; for instance, to expose animals at State fairs and to sell or purchase cattle through loans. In most settings, recent and chronic infection co-exist, with only a small subset of animals being nonreactive to tuberculin due to CMI failure. The farms involved on this study are all commercial beef cow-calf operations in which a permanent herd of cows is kept for producing calves for later sale. In this frame, the most likely scenario will be the progress of the disease to chronic stages, particularly in older cows, such as in this study, with the mean age over 7 years (7.6) and as confirmed by previous results and the official reports of carcass condemnation due to bTB in Para state [33, 37, 38], therefore, antibody responses are likely to be more prevalent (Table 1).

Under the study’s circumstances, no decisions regarding culling animals should be made based only on the SCT test, since the CCT confirmatory test would confirm as positive only 2/59 reactors at the first screening. On the other hand, aiming to avoid additional visits to the farm, veterinarians might adopt the strategy of solely using the CCT test. This strategy applied to our study would result in only two herds and two animals considered to be bTB positive. In turn, considering only the ELISA test, all herds would be classified as bTB positive and 22 CCT negative animals would be diagnosed as bTB positive. Therefore, the sole use of CCT testing would leave behind three infected herds and 22 false-negative animals, which will increase the risk of the disease spreading. The potential of the serological tests to identify non-reactive TST results is being reported and suggests that their application to test non-infected herds would help to increase the performance of the screening strategy in current bTB eradication programs [19, 25, 35].

The application of a parallel testing strategy SCT + ELISA on the numbers of this study would result in no increase on the proportion of infected herds (5/5), however it would result in an increase in the proportion of a positive rate at animal level (75/400). Therefore, in regions with high bTB prevalence and no indemnity for cattle owners (like in this study) it seems to not be reasonable to adopt the above procedure since it notably would increase the false-positive animals, leading to unjustifiable economical loss and ultimately low adherence, or even avoidance, to eradication programs. On the other hand, the parallel strategy of CCT + ELISA would result in an increase of the proportion of herds infected by 60% and at the animal level the increase of positive animals by 5.5%, and these results are compatible to previous studies [17, 25, 35]. Thus, the parallel interpretation of the diagnostic techniques that detect cellular and humoral immune response to detect non-reactive TST animals [17, 25, 34, 35, 39] should be considered according to the local circumstances.

The latent-class Bayesian analysis demonstrated an bTB apparent prevalence of 0.33% which confirm our previous prevalence estimate. Additionally, the Bayesian analysis shows no difference in Sp between CCT testing and ELISA IDEXX™ (Table 5). Thus, the diagnostic power of animals truly negative for bTB is confirmed by the CCT test, standing out as a good confirmatory test for the diagnosis of *M. bovis* infection. Our study design does not allow the assessment of ELISA as an ancillary serial test, trial to test a serial diagnosis to confirm the occurrence of a booster effect in beef cattle under Brazilian conditions. Again, it is important to highlight the limitation of the experimental design adopted due to the absence of the gold standard test (culture) for confirmation of positive animals. The animals included in the study were not destined for slaughter at that time of the study and slaughtering them at that time was not accepted by the owners. In situations like ours where there is no gold standard the Bayesian statistic is recommended. This is the rationale for using the Bayesian statistic to overcome this limitation.

A study to assess the reliability of the combination CFT test (the usual screening test for beef cattle), ELISA in parallel and serial schemes, and isolation of the *M. bovis* by culture (the golden standard) would be of a great value. Adoption of supplemental tests would represent significant logistical improvements on the bTB program, reducing farm visits, CCT test reading errors, time for removing the infection from the herd, and allowing to store serum samples for confirmatory tests for a long period of time. Furthermore, the animals would have to be reunited and handled once, which would be an extra advantage when working with beef, representing a great benefit for the farmers and the veterinarians.

Although the study needs to be extended, circumstantial evidence was obtained to support the recommendation of the adoption of serological tests as supplemental to the traditional TST schemes as an alternative strategy that may contribute to accelerated bTB eradication helping in outbreak management and disease control.

## Conclusions

The official screening test SCT had significantly higher Se than the ELISA IDEXX™, however only 2/59 SCT reactors were confirmed as positive by the CCT test. Therefore, no decisions on animal elimination should be made based only on results of the SCT test.

The ELISA IDEXX™ detected infected animals and herds missed by the CCT test. Parallel use of the CCT test and ELISA increased the Se, the feasibility of screenings for *M. bovis* infection diagnosis, and speed up the cleansing of herds.

The ELISA IDEXX™ can be a supplemental test for *M. bovis* infection detection in regions with no test-and-slaughter routine where the disease usually progress to more advanced stages and antibody responses are likely to be more prevalent.

Results provided evidence to support the validation of a serological test as supplemental to the official TST on the Brazilian eradication program.

## Methods

### Study design

This was a cross-sectional study, designed to assess the feasibility of a supplemental test to improve the detection of bTB in individual animals and herds. A convenience sampling of commercial cow-calf operation farms was conducted through contact with owners from a list provided by the State Ag-defense agency, and those who agreed with the research protocol were included in the study. A total of 400 female Nelore (*Bos taurus indicus*), aged over 24 months, raised on farms in Para State, Brazil, were included. Within each selected farm, a convenient sampling procedure was performed. The animals were selected from a population of 7600 cows, included were cows ranging from 2 to 18 years old (mean 7.06 years), not pregnant, with no history of abortions and with a body condition score of (BSC) > 3. The cows came from five farms located in the following municipalities: Capitao Poco (Farm #1, *n* = 80 of 2500 cows), Garrafao do Norte (Farm #2, n = 80 of 700 cows), and Sao Francisco do Para (Farm #3, n = 80 of 600 cows), in Para’s northeastern region and Castanhal (Farm #4, n = 80 of 1300 cows) and Santa Izabel (Farm #5, n = 80 of 2500 cows) from Belem’s metropolitan mesoregion. The sampling was carried out from March 13 to May 4, 2013.

### Blood collection

Samples were collected from all cows in the experimental group composed of cows from each farm. There was 10.0 mL of blood collected by puncture of the external jugular vein, without excessive tourniquet of the vessel, using siliconized vacutainer tubes without anticoagulant and were properly identified. The samples were centrifuged for 15 min at a speed of 3000 G, then separated by aspiration of the serum, aliquoted in 2 mL Eppendorf microtubes, identified and stored at − 20 °C for subsequent serological testing (ELISA).

### Testing

#### Enzyme-linked Immunosorbent assay – ELISA

The ELISA IDEXX™ *M. bovis* was performed as described previously [16] and according to the manufacturer’s instructions. Shortly after, two microtiter plate wells were used for the positive control, two for the negative control and a blank well to reference the microplate reader. The reading was performed on a TP Reader Basic/Thermoplate microplate reader at a wavelength of 450 nm at an accuracy of ±2 nm and an absorbance resolution of 0.001A at an accuracy of ±0.03A. The results of optical density (OD) provided by the reader were recorded and used to calculate the validation of the test and then the results of the samples according to the specifications of the kit manufacturer.

#### Comparative cervical tuberculin (CCT) skin test

On the same day of blood collection, inoculations of avian (PPDa) and bovine (PPDb) tuberculin were performed intradermally at a dose of 0.1 mL in the cervical region (in places previously demarcated by hair removal) at 15 to 20 cm between the two inoculations. PPDa was inoculated cranially and PPDb was inoculated caudally on the same side of all the animals in the herd to be tested [8]. The skin thickness of the inoculation site was measured using calipers before injection. Test results were determined by the same researcher at 72 h post-injection by measuring the increase in skinfold thickness. Data for analysis of the SCT were obtained from reading only the inoculated PPDb. Interpretations of the test results were made according to the Brazilian standard for screening tests for bTB [32].

#### Statistical analysis

The descriptive statistics of the data comparing the percentages of reactor and non-reactor animals as determined by the ELISA, CCT, and SCT were obtained using the Freq procedure of the Statistical Analysis System (SAS) program (SAS® 9.3, SAS Institute Inc., Cary, North Carolina, US). Further, comparisons were made between the five farms and an interaction term, Exam*Farm.

Inferential statistical analyses were performed using the analysis of variance (ANOVA), with the Glimmix procedure, from the SAS program. Considering that the three exams (ELISA, CCT and SCT) were performed on the same animal, the statistical model was constituted by the exam and farm classificatory variables.

Agreement between diagnostic outcomes (positive vs. negative) from 2 by 2 exams (CCT vs. SCT (PPDb); CCT vs. ELISA; and SCT (PPDb) vs. ELISA) were evaluated by the Kappa coefficient with FREQ procedure of SAS. Interobserver agreement was also evaluated by the weighted Kappa coefficient with Freq procedure of SAS. The significance level of 5% was used for both Least Square Means test (LSMeans) and Kappa analyses.

The Bayesian model is used to assess the sensitivity and specificity of two tests or more where there is no gold standard. As such we compared the CCT and ELISA tests to estimate the Se and Sp using this modelling approach. Samples collected were assumed to originate from a single population given they were drawn from herds in the same geographic area with similar animal health statuses regarding bTB and similar production management standards. A two dependent method tests one population model using the results from the CCT and ELISA. Beta prior distributions for the prevalence, Se and Sp of the CCT and ELISA were chosen according to previous reports. For the analysis, we considered a prevalence of 0.3% ranging from 0.1 to 1.4%, leading to a Beta prior distribution with parameters (2.4; 450). The CCT Se considered was 75% ranging from 60.4 to 85.4%, leading to a Beta prior distribution with parameters (36; 12). The ELISA IDEXX™ Se considered was 90% ranging from 80.6 to 95.1%, leading to a Beta prior distribution with parameters (62.2; 7.8). The CCT Sp considered was 85% ranging from 78.1 to 90.0%, leading to a Beta prior distribution with parameters (120; 22). The ELISA IDEXX™ Sp considered was 99% ranging from 89.6 to 99.8%, leading to a Beta prior distribution with parameters (40.6; 1.4). Combining the prior distributions with the observed data, the estimates for each of the parameters were obtained using the software WinBUGS 1.4 (http://www.mrc-bsu.cam.ac.uk/bugs/winbugs/contents.shtml). The model procedure is described in Additional file 1.

Additionally, the single and parallel interpretations of CCT + ELISA IDEXX™ were done to show the animal and herd-level of Se, as described elsewhere [40].

The comparison between the frequencies of the groups was performed using the test LSMeans of the SAS. The significance level of 5% was used.

## Supplementary Information

**Additional file 1.**

## Data Availability

The authors confirm that all the raw data supporting the findings of this study are available within the article and its supplementary materials.

## Abbreviations

ANOVA: Analysis of variance
bTB: bovine tuberculosis
CCT: Comparative Cervical Tuberculin
CFT: Caudal Fold Tuberculin
CMI: Cell-mediated immune responses
ELISA: Enzyme-linked immunosorbent assay
LSMeans: Least Square Means
*M. bovis*: *Mycobacterium bovis*
OD: Optical density
PPDa: Avian tuberculin
PPDb: Bovine tuberculin
SAS: Statistical Analysis System
Se: Sensitivity
SCT: Single intradermal cervical tuberculin
Sp: Specificity
TB: Tuberculosis
TST: Tuberculin Skin Test
UFRA: Amazon Federal Rural University
US: United States
